# Targeting multiple receptor tyrosine kinases with sitravatinib: A Phase 1b study in advanced renal cell carcinoma and castrate-resistant prostate cancer

**DOI:** 10.1007/s10637-024-01465-9

**Published:** 2024-08-21

**Authors:** Shubham Pant, Byoung Chul Cho, Christos E. Kyriakopoulos, Alexander Spira, Nizar Tannir, Theresa L. Werner, Xiaohong Yan, Saskia Neuteboom, Richard Chao, Sanjay Goel

**Affiliations:** 1 University of Texas MD Anderson Cancer Center, Houston, TX USA; 2 Yonsei Cancer Center, Yonsei University College of Medicine, Seoul, Republic of Korea; 3 University of Wisconsin Carbone Cancer Center, Madison, WI USA; 4 Virginia Cancer Specialists, Fairfax, VA USA; 5 Huntsman Cancer Institute, University of Utah, Salt Lake City, UT USA; 6 Mirati Therapeutics Inc., San Diego, CA USA; 7 Montefiore Medical Center, Bronx, NY USA; 8 Rutgers Cancer Institute, New Brunswick, NJ USA

**Keywords:** Sitravatinib, MGCD516, Renal cell carcinoma, Prostate cancer, Metastatic

## Abstract

Sitravatinib (MGCD516) is an oral inhibitor of several closely related oncogenic tyrosine kinase receptors that include VEGFR-2 (vascular endothelial growth factor receptor-2), AXL, and MET (mesenchymal-epithelial transition). The safety and antitumor activity of sitravatinib are reported in patients from two histologic cohorts (anti-angiogenesis-refractory clear cell renal cell carcinoma [RCC] and castrate-resistant prostate cancer [CRPC] with bone metastases) who participated in a Phase 1/1b study. The patients were enrolled using a 3-stage design that was based on observed objective responses. Objective response rate (ORR) was the primary endpoint. Duration of response, progression-free survival (PFS), overall survival (OS), and safety were also assessed. Overall, 48 patients (RCC n = 38, CRPC n = 10) received ≥ 1 dose of sitravatinib. Both cohorts were heavily pretreated (median number of prior systemic therapies: RCC cohort 3, CRPC cohort 6). In the RCC cohort, ORR was 25.9%, *P* = 0.015 (null hypothesis [ORR ≤ 10%] was rejected). Responses were durable (median duration 13.2 months). Median PFS was 9.5 months and median OS was 30.0 months. No objective responses were seen in the CRPC cohort; median PFS and OS were 5.8 months and 10.1 months, respectively. Across both cohorts, diarrhea (72.9%), fatigue (54.2%), and hypertension (52.1%) were the most frequent all-cause treatment-emergent adverse events (TEAEs). Diarrhea and vomiting (both, 6.3%) were the most frequent serious TEAEs considered related to study treatment. Sitravatinib demonstrated an acceptable safety profile and promising clinical activity in patients with clear cell RCC refractory to prior angiogenesis inhibitor therapy. Strong indicators for clinical activity were not seen in patients with CRPC and bone metastases.

**Clinical trial registration**:ClinicalTrials.gov NCT02219711.

## Introduction

Receptor tyrosine kinase inhibitors (TKIs) targeting aberrant signaling pathways that drive tumorigenesis are a cornerstone treatment for numerous solid tumor types. For example, anti-angiogenic TKIs targeting vascular endothelial growth factor receptor (VEGFR), given with or without an immune checkpoint inhibitor, are the mainstay treatment for metastatic clear cell renal cell carcinoma (RCC)[1, 2] However, resistance to TKIs remains a significant challenge given most patients with RCC who initially respond to VEGFR TKI therapy ultimately relapse [3]. The mechanisms of acquired resistance to TKIs in RCC are complex and, while not fully elucidated, may involve epithelial–mesenchymal transition (EMT), epigenetic modification, lysosomal sequestration, and activating bypass pathways to facilitate the survival of tumor cells in the presence of TKI [3, 4]. Multitargeted receptor TKIs have the potential to increase treatment effectiveness and reduce resistance by addressing multiple dysregulated pathways which promote cancer development and progression [5].

Sitravatinib (MGCD516) is an oral small molecule inhibitor that targets a spectrum of closely related receptor tyrosine kinases (RTKs) implicated in oncogenesis, including but not limited to the TAM family (tyrosine-protein kinase receptor 3 [TYRO3], AXL, MERTK) and split family (VEGFR2, PDGFR [platelet-derived growth factor receptor], KIT) receptors, along with RET and mesenchymal epithelial transition (MET) RTKs [6–8]. Sitravatinib was associated with promising tumor growth suppression in xenograft models of sarcoma, lung cancer, pancreatic cancer, and breast cancer associated with RTK dysfunction, and recently in FLT3-altered models of acute myeloid leukemia [7–9]. The first in-human Phase 1/1b study explored dosing of sitravatinib by assessing dose-limiting toxicities with increasing doses of study medication in patients with a broad range of advanced solid tumors (the most common primary diagnoses were non-small cell lung cancer [NSCLC] and renal cell carcinoma [RCC]) [6]. The pharmacokinetic profile of sitravatinib was evaluated, demonstrating steady and dose-proportional absorption with oral dosing [6]. Safety was also evaluated based on the dose of sitravatinib administered, and preliminary signals of clinical activity were assessed across all patients, irrespective of tumor type, and in the subgroup with NSCLC [6]. The Phase 1b portion of this study aimed to assess the clinical activity of sitravatinib in patients in specific patient groups: those with advanced, unresectable or metastatic tumors with selected histologic diagnoses (anti-angiogenesis agent-refractory clear cell RCC or castrate-resistant prostate cancer [CRPC]), reported here, and in patients with tumors of any type harboring prespecified molecular alterations relevant to the mechanism of action of sitravatinib (to be reported separately) [6].

The rationale for the two histologic diagnoses cohorts included in the Phase 1/1b study centered around an etiology relevant to sitravatinib activity, along with disease burden. For example, RCC resulted in approximately 14,000 deaths and 79,000 new cases in the US in 2022 [10]. Most patients with RCC are diagnosed with clear cell disease, which is characterized by inactivating alterations in von Hippel-Lindau (*VHL*) tumor suppressor gene. This results in increased transcription of hypoxia-inducible transcription factor (HIF) and HIF-targeted genes including VEGF, a key driver of angiogenesis [11, 12]. Since elevated expression of AXL and MET are implicated in resistance to antiangiogenic therapy, simultaneously targeting VEGFR2, MET, and AXL with sitravatinib may target multiple aberrant pathways common in RCC and provide antitumor activity [3, 12, 13]. This hypothesis is consistent with tumor suppressive effects observed with treatment combinations targeting VEGFR2, MET, and AXL in RCC cancer cell lines and mouse xenograft models [12–14].

Prostate cancer is one of the most frequently diagnosed malignancies in men, with bone metastases affecting over 90% of patients with castrate-resistance over the course of their disease [10, 15]. Treatment options are limited for patients with metastatic CRPC after failure of hormonal therapy (abiraterone or enzalutamide) [16]. In the Phase 1/1b study, sitravatinib was evaluated in patients with metastatic CRPC based on the key roles of MET and VEGFR2 in prostate cancer progression, with high expression levels of both RTKs being associated with aggressive disease [17]. Dual targeting of MET and VEGFR2 was shown to suppress growth and osteolysis in prostate cancer bone metastasis models [18, 19]. Furthermore, marked improvements in bone metastases, including complete resolution of some target lesions, were reported with MET and VEGFR2 inhibition in a preliminary study of patients with CRPC [17].

Here, the safety and clinical activity with sitravatinib are reported in patients with anti-angiogenesis-refractory clear cell RCC and CRPC with bone metastases who participated in the first Phase 1/1b study of this agent [6].

## Methods

### Study design

The design of this open-label, Phase 1/1b clinical trial (NCT02219711) was previously reported [6]. In brief, the study comprised periods focused on evaluating the pharmacokinetics (lead-in period), maximum-tolerated dose (Phase 1), and clinical activity (Phase 1b) of sitravatinib in patients with advanced, unresectable or metastatic solid tumors for which standard treatment was not available [6]. Enrollment into the Phase 1b cohorts was based on histologic diagnosis alone: RCC or CRPC (cohorts described here) or by molecular alteration relevant for sitravatinib mechanism of action grouped by tumor histologic diagnosis (to be reported separately). All participants provided written, informed consent.

The patients in the RCC and CRPC Phase 1b cohorts received sitravatinib at an initial starting dose of 150 mg/day (the maximum-tolerated dose established in the Phase 1 cohort [6]). Based on cumulative safety and tolerability data during the study, the starting dose was reduced to 120 mg/day. Dose reductions and interruptions were permitted for adverse events (AEs) assessed as related to study medication. Study treatment was continued at the discretion of the investigator until disease progression, unacceptable toxicity, or withdrawal of consent.

### Study population

Eligible patients were ≥ 18 years and had unresectable clear cell RCC that was refractory to angiogenesis inhibitor therapy, or CRPC with bone metastases. All patients had discontinued their most recent previous therapy ≥ 2 weeks prior to first dose of study treatment and had recovered from any AEs to baseline or Grade 1 (except for alopecia). There were no restrictions on the number of prior lines of therapy. Other key eligibility criteria included life expectancy ≥ 3 months, Eastern Cooperative Oncology Group (ECOG) performance status 0–2, and acceptable hepatic, renal and bone marrow function. Patients were excluded who had symptomatic or uncontrolled brain metastases, significant cardiac abnormalities within the prior 6 months, prolonged QTc interval (> 480 ms), left ventricular ejection fraction (LVEF) < 40%, uncontrolled arterial hypertension, another active cancer (excluding basal cell carcinoma or cervical intra-epithelial neoplasia), recent major surgery (≤ 4 weeks prior to the first dose of study medication), and prior treatment with cabozantinib.

### Study objectives and assessments

The primary objective in the RCC and CRPC Phase 1b cohorts was to assess the clinical activity and safety of sitravatinib. Objective response rate (ORR), in accordance with Response Evaluation Criteria in Solid Tumors (RECIST) v1.1, was the primary efficacy endpoint, with duration of response, progression-free survival (PFS), and overall survival (OS) assessed as secondary endpoints. Tumor at known and suspected disease sites was assessed by computed tomography or magnetic resonance imaging at baseline and 6-week intervals.

The safety assessments included physical examinations, vital sign measurements, electrocardiogram and LVEF measurements, clinical laboratory evaluations, and treatment-emergent adverse events (TEAEs), with severity graded per National Cancer Institute Common Terminology Criteria for Adverse Events (NCI CTCAE) v4.03. Study investigators classified TEAEs as ‘related’ or ‘unrelated’ to study medication (unassigned TEAEs were considered ‘related’ to study medication).

TEAEs were classified as serious if they were life-threatening or resulted in death, required or prolonged hospitalization, resulted in persistent or significant disability, resulted in a congenital abnormality or birth defect, or other event that was assessed as medically important.

### Statistical analysis

The pre-specified null hypothesis was defined as an ORR of ≤ 10%, and an ORR of ≥ 30% was considered interesting. Exact test for single proportion (one-sided alpha = 2.5%) was used to test the null hypothesis (ORR ≤ 10%) against the alternative hypothesis (ORR > 10%). Summaries of ORR and corresponding 95% confidence intervals (CI) were calculated using the binomial proportion confidence interval method. The duration of response (time from first documentation of completed response [CR] or partial response [PR]) to disease progression [PD] per RECIST v1.1, or death due to any cause), PFS (time from first dose of study medication to PD or death due to any cause]) and OS (time from first dose of study medication to death due to any cause) were estimated using Kaplan–Meier methodology. Other data were summarized using descriptive statistics. The primary data cut-off was July 31, 2020, with final analysis performed on October 10, 2022.

Enrollment into the Phase 1b RCC and CRPC cohorts of this study utilized a 3-stage design. Initially, up to 10 patients were planned for each cohort, with enrollment of a further 10 individuals if at least 1 objective response was observed in the initial cohort. If 3–5 responses were observed in these 20 patients, enrollment up to a total of 30 patients was permitted. If there were at least 6 responses in 20 patients, a promising treatment effect warranting further evaluation was indicated and no further patients were enrolled. In contrast, if no responses or fewer than 3 responses were observed in the initial group of 10 or 20 patients, respectively, lack of treatment effect was concluded and no further patients were enrolled.

Safety and clinical activity were analyzed in all patients who received ≥ 1 dose of study medication (modified intent-to-treat [mITT] population). Response was evaluated in the prespecified clinical activity evaluable (CAE) population, which comprised patients who received ≥ 1 cycle of therapy (≥ 80% of assigned dose) and had ≥ 1 post-baseline disease assessment. Response is also reported in the mITT population.

## Results

### Baseline characteristics

Between October 15, 2015 and September 04, 2018, 48 patients (RCC n = 38, CRPC n = 10) were enrolled into the Phase 1b histologic diagnosis cohorts and received ≥ 1 dose of study medication. The median age was 65.0 and 71.5 years in the RCC and CRPC cohorts, respectively, and most patients had ECOG performance status 1 (63.2% and 90.0%, respectively; Table 1). In the RCC cohort almost all patients had disease with clear cell histology (97.4%) and all patients in the CRPC cohort had adenocarcinoma. Both cohorts were heavily pretreated: many had prior surgery (94.7% and 80.0%), prior radiotherapy (44.7% and 70.0%), and the median (range) number of prior systemic therapies was 3 (1–6) and 6 (1–10) in the RCC and CRPC groups, respectively. Also, all patients in the RCC group (n = 38) received prior therapy with an anti-angiogenic agent (Table 1).

**Table 1:** Demographic and disease characteristics (mITT population)

N (%)	RCC (N = 38)	CRPC (N = 10)
Median age (range), years	65.0 (47–80)	71.5 (60–84)
Male	29 (76.3)	10 (100.0)
Ethnicity		
White	26 (68.4)	7 (70.0)
African American	1 (2.6)	2 (20.0)
Asian	10 (26.3)	0
Other	1 (2.6)	1 (10.0)
Histology		
Adenocarcinoma	0	10 (100.0)
Clear cell	37 (97.4)	0
Other	1 (2.6)	0
ECOG performance score		
0	13 (34.2)	1 (10.0)
1	24 (63.2)	9 (90.0)
2	1 (2.6)	0
Prior surgery	36 (94.7)	8 (80.0)
Prior radiotherapy	17 (44.7)	7 (70.0)
Prior systemic therapy	38 (100.0)	10 (100.0)
Median (range) number of prior regimens	3.0 (1–6)	6 (1–10)
GnRH analog	0	9 (90.0)
Anti-androgen	0	8 (80.0)
Abiraterone	0	6 (60.0)
Taxane	0	10 (100.0)
Immunostimulant	0	4 (40.0)
Anti-angiogenic agent		
Pazopanib	22 (57.9)	0
Sunitinib	21 (55.3)	0
Everolimus	13 (34.2)	0
Axitinib	11 (28.9)	0
Temsirolimus	4 (10.5)	0
Bevacizumab	3 (7.9)	0
Lenvatinib	2 (5.3)	0
Cabozantinib	1 (2.6)	0
Sorafenib	1 (2.6)	0

*CRPC* castrate-resistant prostate cancer; *ECOG* Eastern Cooperative Oncology Group; *GnRH* gonadotrophin releasing hormone; *mITT* modified intent-to-treat; *RCC* renal cell carcinoma

### Patient disposition

**Fig. 1:**
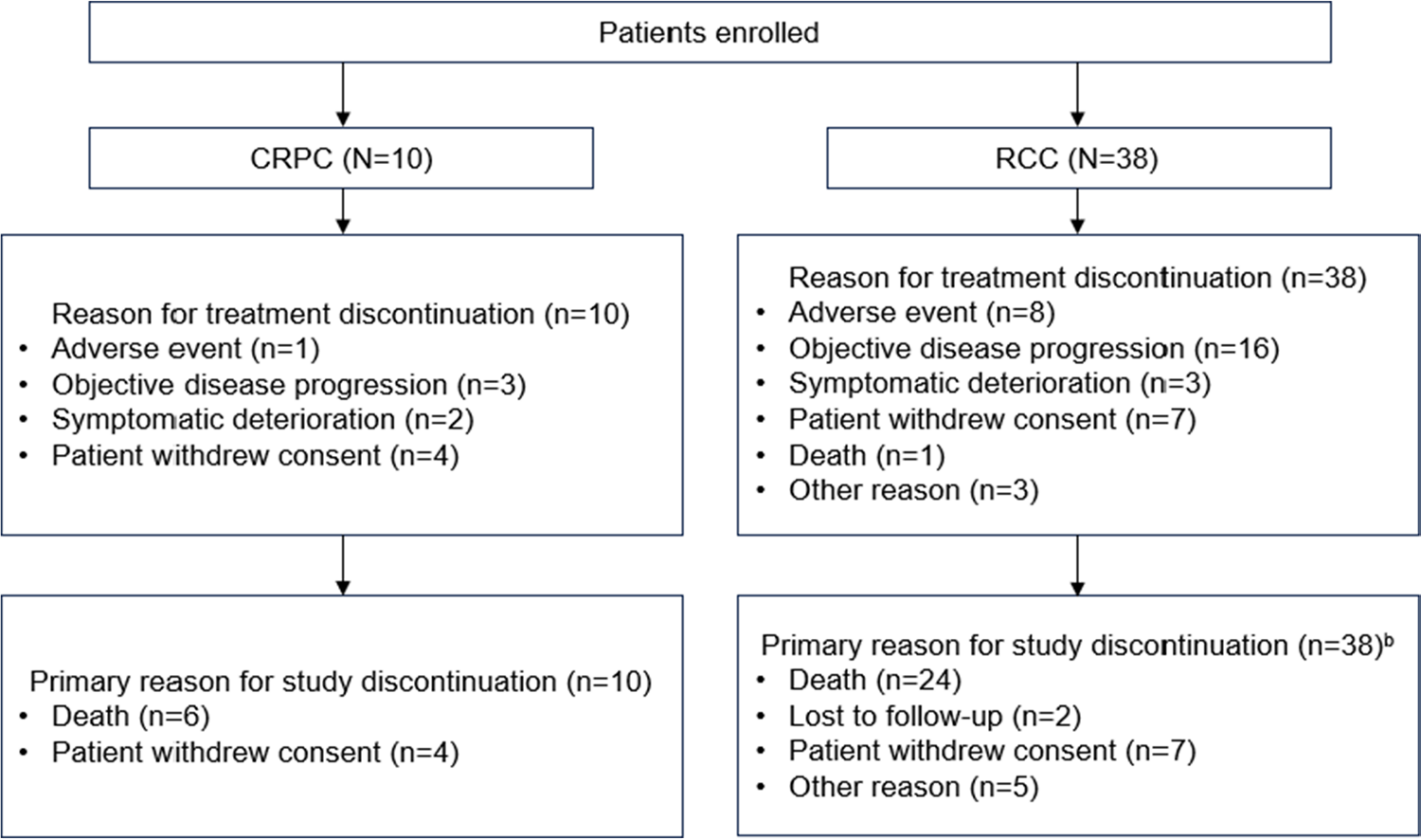
Disposition of patients enrolled in the Phase 1b RCC and CRPC cohorts at final analysis^a^. ^a^October 10, 2022; ^b^Two patients with RCC remained on the study following the primary data cut-off (July 31 2020), and at final analysis (October 10, 2022) n = 1 had discontinued due to disease progression following prior SD, and n = 1 withdrew (patient decision) following BOR. PR CRPC, castrate-resistance prostate cancer; RCC, renal cell carcinoma

Across both cohorts, the most common reasons for treatment discontinuation were disease progression and withdrawal of consent (Fig. 1). Two patients with RCC remained on the study following primary analysis, and at final analysis both had discontinued (n = 1 with best objective response [BOR] of stable disease [SD] discontinued due to PD, and n = 1 with BOR of PR withdrew consent).

### Antitumor activity

The CAE population comprised 27 of 38 patients and 6 of 10 patients enrolled in the RCC and CRPC cohorts, respectively.

**Fig. 2:**
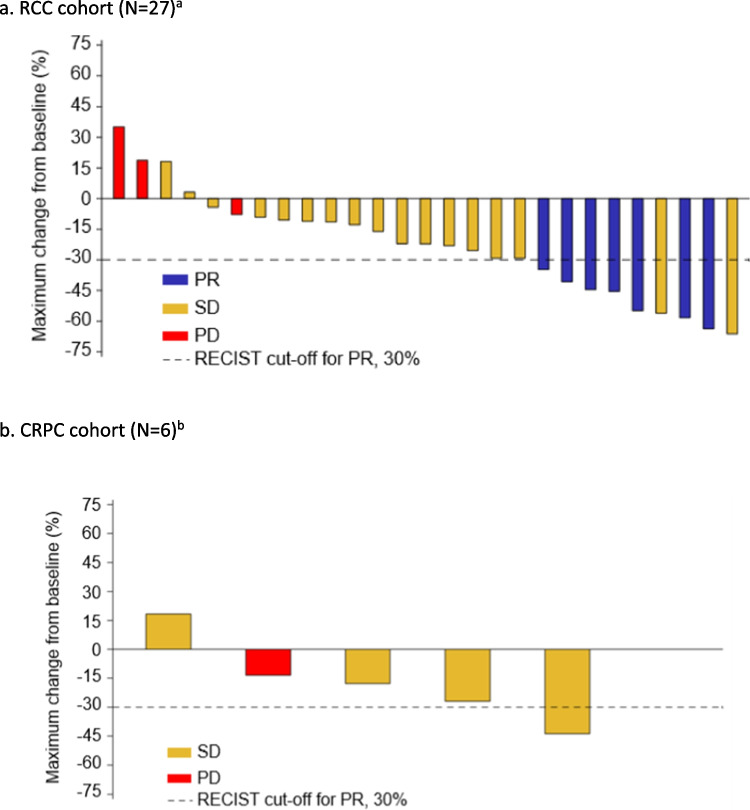
Percentage change in tumor burden (CAE population). a. RCC cohort (N = 27)^a^ b. CRPC cohort (N = 6)^b^. ^a^One additional PR was observed in a patient who received a total dose < 80% in Cycle 1; while this patient did not qualify for the CAE population, they received sufficient study treatment overall to be considered clinically evaluable. ^b^One patient with SD was excluded due to missing change-from-baseline data. CAE, clinical activity evaluable; CRPC, castrate-resistant prostate cancer; PD, disease progression; PR, partial response; RCC, renal cell carcinoma; RECIST, Response Evaluation Criteria in Solid Tumors; SD, stable disease

In the RCC cohort, ORR was 25.9% (PR in 7 of 27 CAE patients), significantly exceeding the null hypothesis of ORR ≤ 10% (vs > 10%) which was rejected (*P* = 0.015). A best response of SD and PD were observed in 17 (63.0%) and 3 (11.1%) patients, respectively. The maximum percentage change in tumor burden is shown in Fig. 2. One additional PR was observed in a patient who received a total dose < 80% in Cycle 1; while this patient did not qualify for the CAE population, they received sufficient study treatment overall to be considered clinically evaluable. Across the 8 patients with PR, the median duration of response was 13.2 months (95% CI 6.8, NE), and the Kaplan–Meier estimate for the proportion of patients with ongoing response at 6 months was 100% (95% CI: 100, 100). Of note, one patient had ongoing PR for 44.1 months before withdrawing from the study and another had SD for 47.8 months until disease progression occurred.

No objective responses were seen in the CRPC cohort. In the CAE population, SD was observed as best response in 83.3% (5 of 6 evaluable patients) and PD in 16.7% (1 of 6 evaluable patients) (Fig. 2). Bone scintigraphy changes consistent with drug activity in bone metastases were noted in one patient.

Objective response outcomes in the mITT populations were similar to the CAE populations. In the RCC cohort, PR was 21.1% (8 of 38 mITT patients, *P* = 0.032), SD was 60.5% (23 of 38 patients) and PD was 10.5% (4 of 38 patients). There were no objective responses in the CRPC mITT population: SD and PD were best responses in 60.0% and 10%, respectively (6 of 10 and 1 of 10 patients, respectively).

**Fig. 3:**
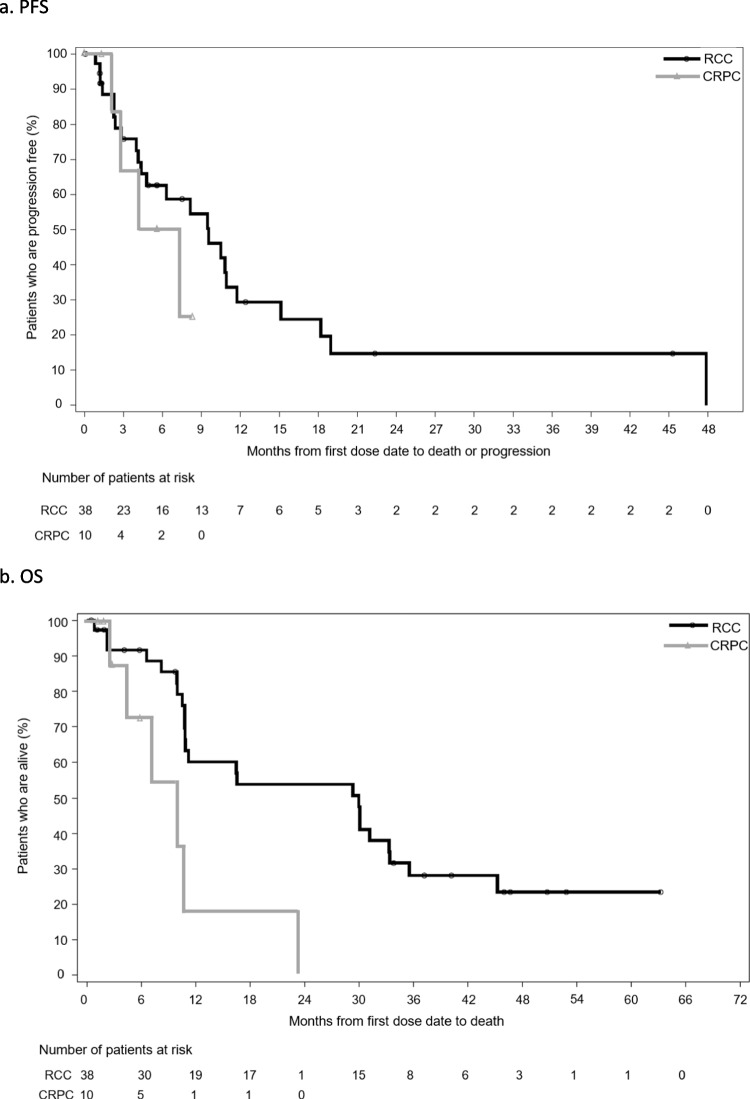
Kaplan–Meier estimates of PFS and OS (mITT population) a. PFS b. OS. CRPC, castrate-resistant prostate cancer; mITT, modified intent to treat; OS, overall survival; PFS, progression-free survival; RCC, renal cell carcinoma

In the RCC cohort (mITT population, n = 38), at final analysis median PFS was 9.5 months (95% CI 4.3, 11.7) and the 6-month PFS estimate was 62.6% (95% CI: 43.5, 76.9) (Fig. 3A). Of the 2 patients who remained on treatment following the primary analysis, PFS was 47.9 months in the patient who developed PD and 45.3 months in the patient who withdrew consent (these patients were censored). At the final analysis, median OS was 30.0 months (95% CI: 10.8, 33.4) and the 12-month OS estimate was 60.1% (95% CI 41.3, 74.6) (Fig. 3B).

In the CRPC cohort (mITT population, n = 10) median PFS was 5.8 months (95% CI 2.1, NE) and the 6-month PFS estimate was 50% (95% CI: 11.1, 80.4) (Fig. 3A). At the final analysis, median OS (mITT) was 10.1 months (95% CI 2.8, 23.2) and the 12-month OS estimate was 18.2% (95% CI: 0.8, 54.5) (Fig. 3B).

### Safety

All patients in the CRPC cohort and most in the RCC cohort (n = 29) received sitravatinib at a starting dose of 150 mg QD, while n = 9 in the RCC cohort were assigned 120 mg QD dose. At the primary data cut-off, patients in the RCC cohort had started a median of 7.5 cycles of study treatment (median 6 cycles for 150 mg QD and 16 cycles for 120 mg QD doses) and median relative dose intensity was 76.7% (77.2% for 150 mg QD and 68.6% for 120 mg QD). The two patients who continued to receive study treatment after primary data cut-off received sitravatinib for a total of 4 years and 3 years 9 months, respectively. The patients in the CRPC cohort started a median of 3.0 cycles of study medication and the median relative dose intensity was 89.9% (starting dose of 150 mg QD).

Overall, the most frequent all-cause TEAEs were diarrhea (72.9%), fatigue (54.2%), hypertension (52.1%), and nausea (50.0%) which occurred at similar frequencies in the RCC and CRPC cohorts (Table 2). These TEAEs were often considered by the study investigator to be related to study treatment: diarrhea 62.5% (RCC 65.8%, CRPC 50.0%) hypertension 47.9% (RCC 50.0%, CRPC 40.0%), fatigue 43.8% (RCC 39.5%, CRPC 60.0%), and nausea 35.4% (RCC 42.1%, CRPC 10.0%).

**Table 2:** Frequent all-cause TEAEs (≥ 10% of patients overall; safety population)^a^

n (%)	RCC (N = 38)		CRPC (N = 10)		All patients (N = 48)	
	All Grade	Grade ≥ 3^b^	All Grade	Grade ≥ 3^b^	All Grade	Grade ≥ 3^b^
Gastrointestinal disorders						
Diarrhea	28 (73.7)	9 (23.7)	7 (70.0)	2 (20.0)	35 (72.9)	11 (22.9)
Nausea	22 (57.9)	6 (15.8)	2 (20.0)	0	24 (50.0)	6 (12.5)
Constipation	18 (47.4)	0	3 (30.0)	0	21 (43.8)	0
Vomiting	16 (42.1)	6 (15.8)	3 (30.0)	1 (10.0)	19 (39.6)	7 (14.6)
Abdominal pain	14 (36.8)	2 (5.3)	0	0	14 (29.2)	2 (4.2)
Stomatitis	6 (15.8)	0	0	0	6 (12.5)	0
General disorders and administration site conditions						
Fatigue	20 (52.6)	3 (7.9)	6 (60.0)	1 (10.0)	26 (54.2)	4 (8.3)
Peripheral edema	6 (15.8)	0	0	0	6 (12.5)	0
Pyrexia	5 (13.2)	0	0	0	5 (10.4)	0
Vascular disorders						
Hypertension	19 (50.0)	7 (18.4)	6 (60.0)	3 (30.0)	25 (52.1)	10 (20.8)
Metabolism and nutrition disorders						
Appetite decreased	13 (34.2)	0	3 (30.0)	0	16 (33.3)	0
Hyperkalemia	11 (28.9)	1 (2.6)	2 (20.0)	0	13 (27.1)	1 (2.1)
Dehydration	9 (23.7)	1 (2.6)	2 (20.0)	2 (20.0)	11 (22.9)	3 (6.3)
Hypomagnesemia	9 (23.7)	0	2 (20.0)	0	11 (22.9)	0
Hyperuricemia	6 (15.8)	0	1 (10.0)	0	7 (14.6)	0
Hyponatremia	6 (15.8)	0	1 (10.0)	1 (10.0)	7 (14.6)	1 (2.1)
Hypercalcemia	5 (13.2)	2 (5.3)	1 (10.0)	0	6 (12.5)	2 (4.2)
Hyperglycemia	6 (15.8)	0	0	0	6 (12.5)	0
Hypoalbuminemia	6 (15.8)	2 (5.3)	0	0	6 (12.5)	2 (4.2)
Hypophosphatemia	5 (13.2)	3 (7.9)	1 (10.0)	1 (10.0)	6 (12.5)	4 (8.3)
Skin and subcutaneous tissue disorders						
Hand–foot syndrome	14 (36.8)	4 (10.5)	2 (20.0)	0	16 (33.3)	4 (8.3)
Rash maculo-papular	6 (15.8)	2 (5.3)	1 (10.0)	0	7 (14.6)	2 (4.2)
Pruritis	4 (10.5)	0	1 (10.0)	0	5 (10.4)	0
Rash	4 (10.5)	0	1 (10.0)	0	5 (10.4)	0
Blood and lymphatic disorders						
Anemia	11 (28.9)	3 (7.9)	5 (50.0)	2 (20.0)	14 (29.2)	5 (10.4)
Investigations						
AST increased	12 (31.6)	1 (2.6)	2 (20.0)	1 (10.0)	14 (29.2)	2 (4.2)
Blood creatinine increased	11 (28.9)	0	3 (30.0)	0	14 (29.2)	0
ALT increased	11 (28.9)	0	1 (10.0)	0	12 (25.0)	0
Weight decreased	8 (21.1)	1 (2.6)	4 (40.0)	0	12 (25.0)	1 (2.1)
Lipase increased	7 (18.4)	5 (13.2)	2 (20.0)	2 (20.0)	9 (18.8)	7 (14.6)
Amylase increased	7 (18.4)	2 (5.3)	1 (10.0)	0	8 (16.7)	2 (4.2)
Platelet count decreased	8 (21.1)	0	0	0	8 (16.7)	0
ALP increased	6 (15.8)	0	1 (10.0)	0	7 (14.6)	0
TSH increased	5 (13.2)	0	0	0	5 (10.4)	0
Endocrine disorders						
Hypothyroidism	11 (28.9)	0	2 (20.0)	0	13 (27.0)	0
Respiratory, thoracic, and mediastinal disorders						
Dysphonia	9 (23.7)	0	4 (40.0)	0	13 (27.1)	0
Cough	7 (18.4)	0	1 (10.0)	0	8 (16.7)	0
Dyspnea	6 (15.8)	2 (5.3)	0	0	6 (12.5)	2 (4.2)
Nasal congestion	3 (7.9)	0	2 (20.0)	0	5 (10.4)	0
Productive cough	4 (10.5)	0	1 (10.0)	0	5 (10.4)	0
Renal and urinary disorders						
Proteinuria	10 (26.3)	2 (5.3)	0	0	10 (20.8)	2 (4.2)
Musculoskeletal and connective tissue disorders						
Back pain	5 (13.2)	1 (2.6)	3 (30.0)	2 (20.0)	8 (16.7)	3 (6.3)
Flank pain	4 (10.5)	0	1 (10.0)	0	5 (10.4)	0
Nervous system disorders						
Headache	7 (18.4)	0	1 (10.0)	0	8 (16.7)	0
Dizziness	6 (15.8)	0	0	0	6 (12.5)	0
Paresthesia	4 (10.5)	0	1 (10.0)	0	5 (10.4)	0
Infections and infestations						
Urinary tract infection	6 (15.8)	1 (2.6)	1 (10.0)	0	7 (14.6)	1 (2.1)

*ALT* alanine aminotransferase, *ALP* alkaline phosphatase, *AST* aspartate aminotransferase, *TSH* thyroid-stimulating hormone

^a^Final analysis (data cut-off October 10, 2022).

^b^NCI CTCAE Grade ≥ 3 corresponds to a severe or life-threatening event

Most AEs were rated as mild or moderate in severity (NCI CTCAE Grade 1 or 2). Across both cohorts, the most frequently reported severe or life-threatening (Grade ≥ 3) AEs of any cause were diarrhea 22.9% (RCC 23.7%, CRPC 20.0%), hypertension 20.8% (RCC 18.4%, CRPC 30.0%), vomiting 14.6% (RCC 15.8%, CRPC 10.0%), and lipase increased 14.6% (RCC 13.2%, CRPC 20.0%) (Table 2).

Across both cohorts the most common serious TEAEs were gastrointestinal, including vomiting 10.4% (RCC n = 5 [13.2%]), diarrhea 6.3% (RCC n = 1 [2.6%], CRPC n = 2 [20.0%]), and nausea 6.3% (RCC n = 3 [7.9%]). Serious TEAEs considered by the investigator to be related to study treatment that occurred in ≥ 2 patients across both cohorts were also predominantly gastrointestinal: diarrhea (n = 3, 6.3%), vomiting (n = 3, 6.3%), nausea (n = 2, 4.2%), and fatigue (n = 2, 4.2%).

Overall, three patients died due to TEAEs of cardiac arrest, gastrointestinal hemorrhage, and sepsis (each, n = 1 in RCC cohort); cardiac arrest was the only death considered by the investigator to be related to study treatment. This event occurred in a male patient (77 years) who received sitravatinib 150 mg/day. The patient had a history of RCC, splenic and mesenteric vein thrombosis, hyperlipidemia, and hypothyroidism and was previously treated with sunitinib, pazopanib, nivolumab, and an experimental glutaminase inhibitor. The patient died on Day 25 after developing chest pain that was treated with epinephrine and cardioversion. One additional death was reported as a TEAE in the RCC cohort and was attributed to disease progression.

## Discussion

The antitumor activity and safety of sitravatinib, a potent inhibitor of oncogenic TAM and split kinase receptor families, are reported in two histologic diagnosis cohorts from a Phase 1b study. Patients with clear cell RCC refractory to prior angiogenesis inhibitor therapy and CRPC with bone metastases, two diseases with RTK targets of sitravatinib commonly implicated in their etiology, were enrolled to provide insight into sitravatinib clinical activity based on clinical diagnosis alone. Patients with solid tumors containing molecular alterations relevant to the mechanism of action of sitravatinib who were also enrolled using a basket cohort approach are reported separately.

As previously described, across all evaluable Phase 1b patients, which included a broad range of solid tumor types, the clinical activity of sitravatinib was modest (ORR of 11.8%), as was also observed in a subgroup of participants with NSCLC (ORR 13.2%) [6]. In contrast, in this population with clear cell RCC refractory to prior antiangiogenic therapy who were not molecularly selected, sitravatinib was associated with an ORR of 25.9%, including two patients who remained progression-free for over 45 months. Median PFS was 9.5 months and median OS was 30 months. It is noteworthy that this promising clinical activity was observed in patients with RCC that was refractory to prior VEGFR inhibitors. This suggests simultaneous targeting VEGFR2, MET, and AXL with sitravatinib may overcome potential MET-mediated antiangiogenic treatment resistance in some patients. This hypothesis is supported by the elevated expression of AXL and MET reported in RCC cell lines chronically treated with sunitinib, and inhibiting AXL and MET led to tumor size reductions in xenograft models with acquired resistance to sunitinib [13].

During the course of this study, cabozantinib, a TKI with TAM and SPLIT family receptor targets in common with sitravatinib, as well as FLT3, ROS1 and TIE-2, was approved for patients with VEGFR-TKI-refractory RCC, further supporting the rationale for sitravatinib in this setting [20]. This approval was based on the METEOR study in which cabozantinib demonstrated greater PFS (7.4 vs 3.9 months), OS (21.4 vs 16.5 months), and ORR (17% vs 3%) compared with everolimus [21]. While the clinical activity of sitravatinib in antiangiogenic agent-refractory RCC observed in our study appears promising in light of these data, particularly considering that most patients in METEOR had received only one prior VEGFR TKI, direct cross-study comparison is not recommended due to variances in design, including lack of a comparator arm in our study, and study populations.

The clinical activity of sitravatinib in CRPC and bone metastases was less promising than observed in participants with clear cell RCC. As no objective responses were seen in the initial cohort of 10 patients (n = 6 were evaluable for response), based on the 3-stage design of this study enrollment was halted. Median PFS was 5.8 months and median OS was 10.1 months. In other TKI studies, targeting angiogenesis in patients with metastatic CRPC also failed to impact OS despite convincing clinical activity in other tumor types [22]. For example, despite preventing the progression of prostate cancer in bone and soft tissue in xenograft models and promising clinical activity in a Phase 2 study, cabozantinib did not meet the primary endpoint of OS or pain improvement in pretreated patients with metastatic CRPC in the Phase 3 COMET-1 and COMET-2 studies, respectively [23–26]. These findings highlight the challenges of tumor microenvironment heterogeneity associated with CRPC and the need for biomarker-driven screening [22].

Sitravatinib had a manageable safety profile in patients with advanced RCC and CRPC who have received several lines of prior systemic therapy. Overall, 18.8% of patients experienced AEs that led to discontinuation of study treatment. Most AEs considered related to sitravatinib tended to be gastrointestinal events (diarrhea 62.5% and nausea 35.4%), hypertension (47.9%), and fatigue (43.8%). This safety profile is expected, being consistent with on-target inhibition and the safety profile reported for cabozatinib [21, 23]. It is noteworthy that most patients (39 of 48) received sitravatinib at the previously defined MTD of 150 mg QD rather than 120 mg daily (9 of 48: 120 mg daily was recommended for further evaluation based on ongoing observations of a lower incidence of serious AEs and severe treatment-related AEs) [6]. The tolerability profile of sitravatinib would likely been improved had all Phase 1b patients received the lower dose. It is unlikely clinical activity in the RCC cohort would have been impaired if all patients had received the 120 mg daily dose of stitravatinib, given concentration-dependent modulation of plasma VEGF-A and soluble-VEGF-R2 levels was consistent with effective targeting of the VEGF-R family at both doses of study treatment [6]. However, further evaluation would be required.

Since this onset of this study, checkpoint inhibitors have become the standard of care for metastatic clear cell RCC, and combination therapy with a VEGFR TKI is among the recommended first-line treatment approaches [1]. However, while inhibiting VEGFR and programmed death-ligand 1 pathways has improved survival outcomes, some patients do not respond to this treatment and many develop resistance [27]. Targeting TAM and split family RTKs with sitravatinib has the potential to augment antitumor immune responses. In preclinical studies sitravatinib reversed an immunosuppressive tumor microenvironment by mechanisms including increased M1/M2-polarized macrophage ratio, and reduced myeloid-derived suppressor cells and regulatory T-cells [8, 28]. Furthermore, in xenograft models of lung and breast cancer, combining sitravatinib with immune checkpoint inhibition enhanced antitumor effects [8].

A subsequent single-arm, dose-finding study of sitravatinib in combination with nivolumab in patients with clear cell RCC refractory to prior antiangiogenic therapy also reported encouraging clinical activity and correlative immune effects, with sitravatinib reducing immune-suppressive myeloid cells in the TME [29]. Of note, the clinical activity of sitravatinib plus nivolumab (ORR 35.7% and median PFS 11.7 months) exceeded that of single-agent nivolumab reported in the same setting in CheckMate 025 [30]. Based on these data, sitravatinib in combination with checkpoint inhibition is being further investigated, including two Phase 3 trials in NSCLC (NCT03906071, NCT04921358). Clinical data from other compounds also support the rationale for targeting TAM and split RTKs to enhance immune response to checkpoint inhibition. Indeed, cabozantinib plus nivolumab was approved as a first-line treatment for advanced clear cell RCC after demonstrating the superior efficacy to sunitinib monotherapy, and cabozantinib plus atezolizumab showed promising antitumor activity (ORR 23%) in patients with metastatic CRPC refractory to hormone therapy and soft tissue progression in an open-label Phase 1b [31, 32].

In summary, single-agent sitravatinib had an acceptable safety profile and demonstrated promising clinical activity in patients with molecularly unselected clear cell RCC that was refractory to prior angiogenesis inhibitor therapy. In contrast, strong indictors for clinical activity were not seen in patients with CRPC and bone metastases. Emerging data indicate that the potential utility of sitravatinib may be further enhanced by combination treatment with an immune checkpoint inhibitor, and this is being investigated in on-going studies.

## Data Availability

Mirati Therapeutics Inc. are committed to patient care, as well as advancing scientific understanding and enabling the scientific community to learn from and build upon the research we have undertaken. We will honor legitimate requests for our clinical trial data from qualified researchers / investigators for methodologically sound research. Sharing clinical trial data, clinical study reports, study protocols, and statistical analysis plans for this study is subject to protecting patient privacy and respect for the patient’s informed consent. In general, data will be available for specific requests approximately 2 years after clinical trial completion from our in-scope interventional trials. Email medinfo@mirati.com for further information relating to data sharing collaborations.

## References

[1] Rathmell WK, Rumble RB, Van Veldhuizen PJ et al (2022) Management of metastatic clear cell renal cell carcinoma: ASCO Guideline. J Clin Oncol 40:2957–2995. 10.1200/JCO.22.00868 35728020 10.1200/JCO.22.00868

[2] Escudier B, Porta C, Schmidinger M et al (2019) Renal cell carcinoma: ESMO Clinical Practice Guidelines for diagnosis, treatment and follow-up. Ann Oncol 30:706–720. 10.1093/annonc/mdz056 30788497 10.1093/annonc/mdz056

[3] Sharma R, Kadife E, Myers M et al (2021) Determinants of resistance to VEGF-TKI and immune checkpoint inhibitors in metastatic renal cell carcinoma. J Exp Clin Cancer Res 40:186. 10.1186/s13046-021-01961-3 34099013 10.1186/s13046-021-01961-3 PMC8183071

[4] Sekino Y, Teishima J, Liang G, Hinata N (2022) Molecular mechanisms of resistance to tyrosine kinase inhibitor in clear cell renal cell carcinoma. Int J Urol 29:1419–1428. 10.1111/iju.15042 36122306 10.1111/iju.15042 PMC10087189

[5] Pottier C, Fresnais M, Gilon M et al. (2020). Tyrosine kinase inhibitors in cancer: breakthrough and challenges of targeted therapy. Cancers (Basel) 12. 10.3390/cancers12030731. 10.3390/cancers12030731 PMC7140093 32244867

[6] Bauer T, Cho BC, Heist R et al (2022) First-in-human phase 1/1b study to evaluate sitravatinib in patients with advanced solid tumors. Invest New Drugs 40:990–1000. 10.1007/s10637-022-01274-y 35767205 10.1007/s10637-022-01274-y PMC9395446

[7] Patwardhan PP, Ivy KS, Musi E et al. (2016). Significant blockade of multiple receptor tyrosine kinases by MGCD516 (sitravatinib), a novel small molecule inhibitor, shows potent anti-tumor activity in preclinical models of sarcoma. Oncotarget 7:4093–4109. 10.18632/oncotarget.6547. 10.18632/oncotarget.6547 PMC4826192 26675259

[8] Du W, Huang H, Sorrelle N, Brekken RA (2018). Sitravatinib potentiates immune checkpoint blockade in refractory cancer models. JCI Insight 3. 10.1172/jci.insight.124184. 10.1172/jci.insight.124184 PMC6238734 30385724

[9] Zhang Y, Wang P, Wang Y, Shen Y (2023) Sitravatinib as a potent FLT3 inhibitor can overcome gilteritinib resistance in acute myeloid leukemia. Biomark Res 11:8. 10.1186/s40364-022-00447-4 36691065 10.1186/s40364-022-00447-4 PMC9872318

[10] Siegel RL, Miller KD, Fuchs HE, Jemal A (2022) Cancer statistics, 2022. CA Cancer J Clin 72:7–33. 10.3322/caac.21708 35020204 10.3322/caac.21708

[11] Chen YW, Rini BI, Beckermann KE (2022). Emerging targets in clear cell renal cell carcinoma. Cancers (Basel) 14. 10.3390/cancers14194843. 10.3390/cancers14194843 PMC9561986 36230766

[12] Ciamporcero E, Miles KM, Adelaiye R et al (2015) Combination strategy targeting VEGF and HGF/c-met in human renal cell carcinoma models. Mol Cancer Ther 14:101–110. 10.1158/1535-7163.MCT-14-0094 25381264 10.1158/1535-7163.MCT-14-0094 PMC4297225

[13] Zhou L, Liu XD, Sun M et al (2016) Targeting MET and AXL overcomes resistance to sunitinib therapy in renal cell carcinoma. Oncogene 35:2687–2697. 10.1038/onc.2015.343 26364599 10.1038/onc.2015.343 PMC4791213

[14] Wang X, Solban N, Khanna P et al. (2016). Inhibition of ALK1 signaling with dalantercept combined with VEGFR TKI leads to tumor stasis in renal cell carcinoma. Oncotarget 7:41857–41869. 10.18632/oncotarget.9621. 10.18632/oncotarget.9621 PMC5173101 27248821

[15] Den RB, George D, Pieczonka C, McNamara M (2019) Ra-223 treatment for bone metastases in castrate-resistant prostate cancer: Practical management issues for patient selection. Am J Clin Oncol 42:399–406. 10.1097/COC.0000000000000528 30844849 10.1097/COC.0000000000000528 PMC6445613

[16] Parker C, Castro E, Fizazi K et al (2020) Prostate cancer: ESMO Clinical Practice Guidelines for diagnosis, treatment and follow-up. Ann Oncol 31:1119–1134. 10.1016/j.annonc.2020.06.011 32593798 10.1016/j.annonc.2020.06.011

[17] Lee RJ, Smith MR (2013) Targeting MET and vascular endothelial growth factor receptor signaling in castration-resistant prostate cancer. Cancer J 19:90–98. 10.1097/PPO.0b013e318281e280 23337762 10.1097/PPO.0b013e318281e280 PMC3683553

[18] Lee C, Whang YM, Campbell P et al (2018) Dual targeting c-met and VEGFR2 in osteoblasts suppresses growth and osteolysis of prostate cancer bone metastasis. Cancer Lett 414:205–213. 10.1016/j.canlet.2017.11.016 29174801 10.1016/j.canlet.2017.11.016

[19] Eswaraka J, Giddabasappa A, Han G et al (2014) Axitinib and crizotinib combination therapy inhibits bone loss in a mouse model of castration resistant prostate cancer. BMC Cancer 14:742. 10.1186/1471-2407-14-742 25277255 10.1186/1471-2407-14-742 PMC4190397

[20] Osanto S, van der Hulle T (2018) Cabozantinib in the treatment of advanced renal cell carcinoma in adults following prior vascular endothelial growth factor targeted therapy: clinical trial evidence and experience. Ther Adv Urol 10:109–123. 10.1177/1756287217748867 29662541 10.1177/1756287217748867 PMC5896860

[21] Choueiri TK, Escudier B, Powles T et al (2016) Cabozantinib versus everolimus in advanced renal cell carcinoma (METEOR): final results from a randomised, open-label, phase 3 trial. Lancet Oncol 17:917–927. 10.1016/S1470-2045(16)30107-3 27279544 10.1016/S1470-2045(16)30107-3

[22] Solimando AG, Kalogirou C, Krebs M (2022) Angiogenesis as therapeutic target in metastatic prostate cancer: narrowing the gap between bench and bedside. Front Immunol 13:842038. 10.3389/fimmu.2022.842038 35222436 10.3389/fimmu.2022.842038 PMC8866833

[23] Basch EM, Scholz M, de Bono JS et al (2019) Cabozantinib versus mitoxantrone-prednisone in symptomatic metastatic castration-resistant prostate cancer: a randomized phase 3 trial with a primary pain endpoint. Eur Urol 75:929–937. 10.1016/j.eururo.2018.11.033 30528222 10.1016/j.eururo.2018.11.033 PMC6876845

[24] Smith M, De Bono J, Sternberg C et al (2016) Phase III study of cabozantinib in previously treated metastatic castration-resistant prostate cancer: COMET-1. J Clin Oncol 34:3005–3013. 10.1200/JCO.2015.65.5597 27400947 10.1200/JCO.2015.65.5597

[25] Dai J, Zhang H, Karatsinides A et al (2014) Cabozantinib inhibits prostate cancer growth and prevents tumor-induced bone lesions. Clin Cancer Res 20:617–630. 10.1158/1078-0432.CCR-13-0839 24097861 10.1158/1078-0432.CCR-13-0839 PMC3946460

[26] Smith DC, Smith MR, Sweeney C et al (2013) Cabozantinib in patients with advanced prostate cancer: results of a phase II randomized discontinuation trial. J Clin Oncol 31:412–419. 10.1200/JCO.2012.45.0494 23169517 10.1200/JCO.2012.45.0494 PMC4110249

[27] Ballesteros PA, Chamorro J, Roman-Gil MS et al. (2021). Molecular mechanisms of resistance to immunotherapy and antiangiogenic treatments in clear cell renal cell carcinoma. Cancers (Basel) 13. 10.3390/cancers13235981. 10.3390/cancers13235981 PMC8656474 34885091

[28] Msaouel P, Genovese G, Gao J et al (2021) TAM kinase inhibition and immune checkpoint blockade: a winning combination in cancer treatment? Expert Opin Ther Targets 25:141–151. 10.1080/14728222.2021.1869212 33356674 10.1080/14728222.2021.1869212 PMC12269679

[29] Msaouel P, Goswami S, Thall PF et al. (2022). A phase 1–2 trial of sitravatinib and nivolumab in clear cell renal cell carcinoma following progression on antiangiogenic therapy. Sci Transl Med 14:eabm6420. 10.1126/scitranslmed.abm6420. 10.1126/scitranslmed.abm6420 PMC11924199 35442707

[30] Motzer RJ, Escudier B, McDermott DF et al (2015) Nivolumab versus everolimus in advanced renal-cell carcinoma. N Engl J Med 373:1803–1813. 10.1056/NEJMoa1510665 26406148 10.1056/NEJMoa1510665 PMC5719487

[31] Choueiri TK, Powles T, Burotto M et al (2021) Nivolumab plus cabozantinib versus sunitinib for advanced renal-cell carcinoma. N Engl J Med 384:829–841. 10.1056/NEJMoa2026982 33657295 10.1056/NEJMoa2026982 PMC8436591

[32] Agarwal N, McGregor B, Maughan BL et al (2022) Cabozantinib in combination with atezolizumab in patients with metastatic castration-resistant prostate cancer: results from an expansion cohort of a multicentre, open-label, phase 1b trial (COSMIC-021). Lancet Oncol 23:899–909. 10.1016/S1470-2045(22)00278-9 35690072 10.1016/S1470-2045(22)00278-9

